# Supplementary material for: Nuclear genetic background influences the phenotype of the Drosophila tko25t mitochondrial protein-synthesis mutant
Source: G3 (Bethesda). 2023 Apr 5;13(6):jkad078. doi: 10.1093/g3journal/jkad078 (PMC10234395; doi:10.1093/g3journal/jkad078)
Supplement: jkad078_Supplementary_Data [file jkad078_supplementary_data.zip › Figure S1.pdf]

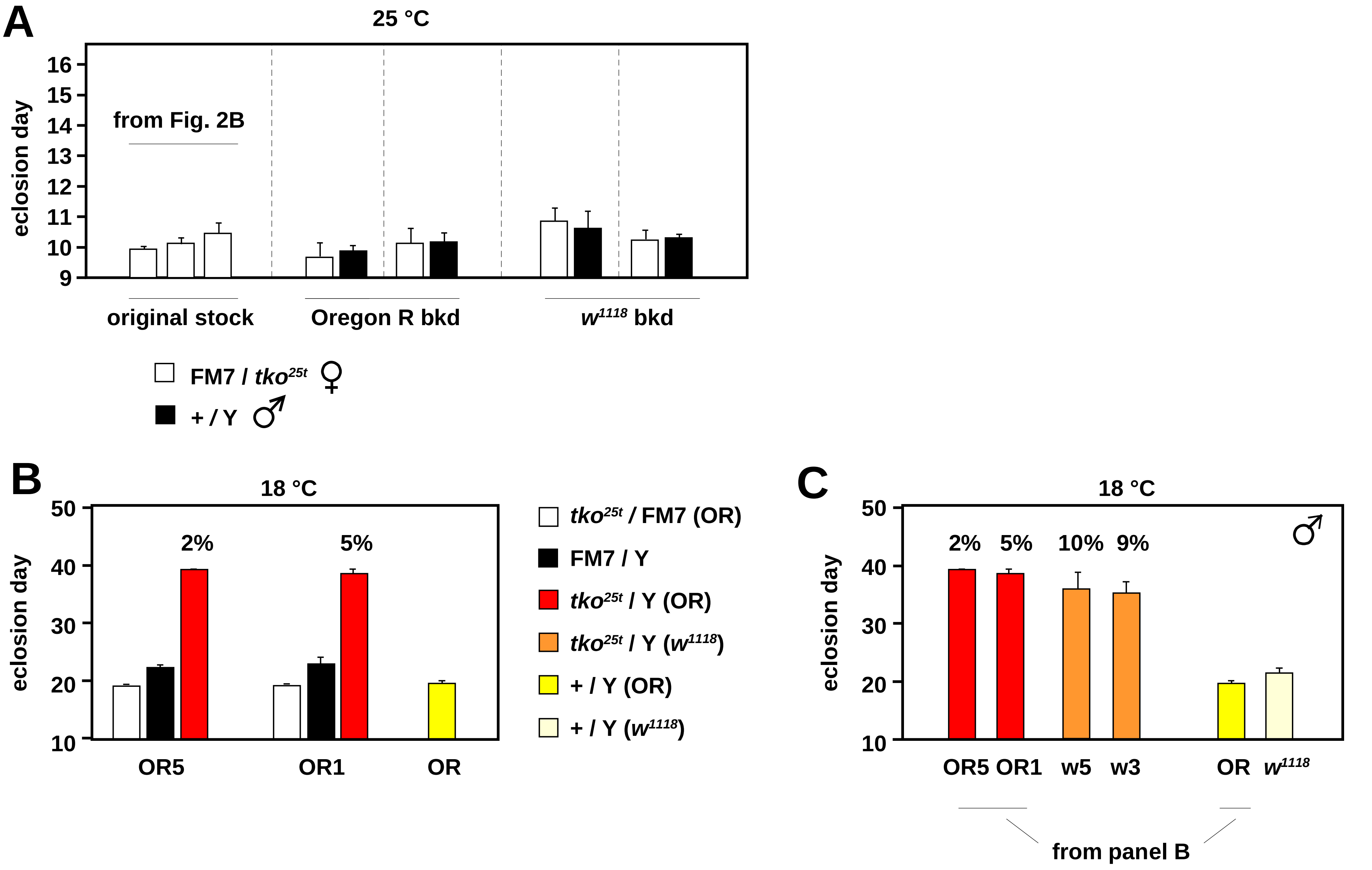

FOR LEGEND SEE NEXT PAGE

Figure S1
Supplementary data on eclosion timing of backcrossed strains

(A) Eclosion times (means ± SD) for control flies from the original *tko*^{25t} stock and *w*^{1118} and Oregon R backgrounds, as indicated. Dashed vertical lines demarcate separate experiments. Despite minor variations from experiment to experiment, the eclosion times for wild-type males were the same as for *tko*^{25t} / FM7 heterozygous females of the given background. (B) Eclosion times (means ± SD) for flies of the indicated genotypes, cultured at 18 °C, from the Oregon R (OR) background. Experimental cross: *tko*^{25t} / FM7 x FM7 / Y. Note that the FM7 balancer itself confers a modest developmental delay (FM7 female homozygotes not shown, for clarity). Control cross conducted in parallel. % figures above the bars indicate the % of progeny of that phenotype class (expected frequency = 25%). (C) Eclosion times (means ± SD) for male flies of the indicated genotypes, cultured at 18 °C, from the two backgrounds, Oregon R (OR) and *w*^{1118}. Data for OR reproduced from (B).
